# Publisher Correction: Perovskites fabricated on textured silicon surfaces for tandem solar cells

**DOI:** 10.1038/s42004-020-0317-y

**Published:** 2020-05-22

**Authors:** Sang-Won Lee, Soohyun Bae, Jae-Keun Hwang, Wonkyu Lee, Solhee Lee, Ji Yeon Hyun, Kyungjin Cho, Seongtak Kim, Friedemann D. Heinz, Sung Bin Choi, Dongjin Choi, Dongkyun Kang, Jeewoong Yang, Sujeong Jeong, Se Jin Park, Martin C. Schubert, Stefan Glunz, Won Mok Kim, Yoonmook Kang, Hae-Seok Lee, Donghwan Kim

**Affiliations:** 1 Department of Materials Science and Engineering, Korea University, Seoul, 136-713 Republic of Korea; 2 Gangwon Regional Division, Korea Institute of Industrial Technology, Gangwon-Do, 210-340 Republic of Korea; 3 Laboratory for Photovoltaic Energy Conversion, Department of Sustainable Systems Engineering (INATECH), University Freiburg, Emmy-Noether Strasse 2, 79110 Freiburg, Germany; 4 Korea Institute of Science and Technology (KIST), Seoul, 02792 Republic of Korea; 5 Fraunhofer Institute for Solar Energy Systems ISE, Freiburg, 79110 Germany; 6 Laboratory for Photovoltaic Energy Conversion, University Freiburg, Freiburg, 79110 Germany; 7 KU·KIST Green School, Graduate School of Energy and Environment, Korea University, Seoul, 136-713 Republic of Korea

**Keywords:** Solar cells, Synthesis and processing, Devices for energy harvesting

Correction to: *Communications Chemistry* 10.1038/s42004-020-0283-4, published online 25 March 2020.

The original version of this Article incorrectly designated Yoonmook Kang as a corresponding author and listed their e-mail address in error. This has now been corrected in the PDF and HTML versions of the Article.

