# Supplementary material for: Perovskites fabricated on textured silicon surfaces for tandem solar cells
Source: Commun Chem. 2020 Mar 25;3:37. doi: 10.1038/s42004-020-0283-4 (PMC9814648; doi:10.1038/s42004-020-0283-4)
Supplement: Supplementary file 1 — Supplementary Information [file 42004_2020_283_MOESM1_ESM.pdf]

Supporting Information

Perovskites Fabricated on Textured Silicon Surfaces for Tandem Solar Cells

Sang-Won Lee, et al.

Supplementary Table 1. List of studies on perovskite/silicon two-terminal tandem solar cells. In the process column, 2.S, 1.S, and 2.H indicate 2-step solution, 1-step solution, and 2-step hybrid methods, respectively. The methods are summarized in Supplementary Table 2.

Published Month	Si texture	ARC	V_{oc} (mV)	J_{sc} (mA/cm^2)	FF (%)	PCE (%)	Steady-state or certified PCE (%)	Area (cm^2)	Process	Reference
2015.03	Rear	LiF	1580	11.5	75.0	N/P	13.7	1.00	2.S	MIT/Stanford [1]
2015.10	-	-	1785	14.0	79.5	19.9 (R)	18.1	0.16	1.S	HZB/EPFL [2]
2015.12	-	ARF	1690	15.9	77.6	20.9 (R)	21.2	0.17	2.H	EPFL [3]
	-	ARF	1701	16.1	70.1	19.2 (R)	19.2	1.22	2.H	
2016.07	Rear	ARF	1718	16.4	70.0	19.7 (R)	20.5	1.43	2.H	EPFL [4]
2016.12	-	ARF	1677	15.3	67.6	17.4 (R)	16.3	1.43	1.S	EPFL [5]
2017.02	Rear	LiF	1650	18.1	79.0	23.6	23.6	1.00	1.S	Stanford [6]
2017.10	Rear	MgF_2	1751	16.8	77.5	22.8 (R)	22.0	0.25	2.H	EPFL [7]
	Rear	MgF_2	1777	16.5	74.4	21.8 (R)	21.2	1.43	2.H	
	Rear	MgF_2	1769	16.5	65.4	19.1 (R)	18.0	12.96	2.H	
2017.10	Rear	Silicone foil	1750	17.6	72.2	22.3 (R)	22.5	1.00	1.S	ANU [8]
2017.10	-	-	1703	15.3	79.2	20.6 (R)	18.0	0.03	1.S	PKU [9]
2017.12	-	-	1710	15.5	71.0	18.8 (R)	N/P	0.13	1.S	NKU [10]
2018.06	Front/Rear	MgF_2	1788	19.5	73.1	25.5 (R)	25.2	1.42	2.H	EPFL[11]
2018.06	N/P	N/P	1793	19.0	73.8	N/P	25.2	1.088	N/P	HZB/Oxford PV[12]
2018.06	N/P	N/P	1810	20.6	73.4	27.3	26.7	1.00	N/P	Oxford PV [13]
2018.06	-	PDMS	1676	16.1	78.0	21.0 (R)	20.5	4	2.S	UNSW [14]
	-	PDMS	1658	15.6	68.0	17.6 (R)	17.1	16	2.S	

2018.08	Rear	PDMS	1740	16.2	78.0	21.9 (R)	21.8	16	1.S	UNSW [15]
2018.08	Rear	PDMS	1770	18.4	77.0	25.0 (R)	N/P	1	1.S	Stanford [16]
2018.10	Rear	MgF_2	1800	17.8	79.4	25.4	N/P	0.04	1.S	Nebraska-Lincoln [17]
2018.10	Rear	Lacquer	1760	18.5	78.5	25.5 (R)	N/P	0.77	1.S	HZB [18]
2018.10	-	-	1660	16.5	81.0	22.2	N/P	0.06	2.S	PKU [19]
2018.10	-	-	1780	17.1	74.0	22.8	N/P	0.13	1.S	NKU [20]
2018.11	-	PDMS	1750	16.9	74.2	21.9	N/P	0.13	1.S	NKU [21]
2018.12	N/P	N/P	1801	19.8	78.7	N/P	28.0	1.00	N/P	Oxford PV [22]
2018.12	Rear	Silicone foil	1763	17.8	78.1	24.5 (R)	24.1	1.00	1.S	ANU [23]
2018.12	Rear	Moth-eye film	1670	18.3	77.0	23.5 (R)	23.4	1.00	1.S	SNU [24]
2019.01	-	-	1830	15.9	70.0	20.4	N/P	0.24	1.S	NKU [25]
2019.02	Rear	N/P	1792	19.0	74.6	25.4 (R)	25.2	1.09	1.S	Oxford [26]
2019.03	Front	MgF_2	1741	19.5	74.7	25.4	25.1	1.42	2.H	EPFL [27]
2019.03	-	LiF	1645	16.1	79.9	21.2	N/P	0.27	1.S	UNIST/KIST [28]
2019.04	-	MgF_2	1735	18.5	75.0	24.5(R)	24.0	1.43	1.S	EPFL[29]
	-	MgF_2	1723	17.5	75.0	22.6(R)	22.6	57.4	1.S	
2019.05	Rear	LiF	1780	17.8	78.6	25.0	25.0	0.77	1.S	HZB [30]
2019.08	-	MgF_2/Silica	1780	14.7	80.4		21.0	0.15	1.S	FAU [31]
2019.08	Front	PDMS	1590	15.5	79.0	19.4	19.4	0.25	1.S	UNIST/Shinsung E&G [32]
2019.10	Rear	PDMS	1732	16.5	81.0	23.0	23.0	4.0	1.S	UNSW [33]
(Triple Junction) 2018.08	Front/Rear	MgF_2	2688	7.7	68.0	14.0 (R)	13.2	1.42	2.H	EPFL [34]

Supplementary Table 2. Representative perovskite fabrication processes.

	One-step	Two-step
Solution	Spin coating [35, 36]<br>Drop casting [37]<br>Soft-cover deposition [38]<br>Blade [39, 40]<br>Slot die [41]<br>Spray coating [42]	Sequential deposition [43]<br>Inter diffusion spin-coating [44]
Dry	Coevaporation [45, 46]<br>Chemical vapor deposition (CVD) [47]	Sequential vapor deposition (SVD) [48]<br>Precursor evaporation + CVD [49]<br>Precursor sputtering + vapor annealing [50]
Hybrid (solution + dry)	-	Precursor spin-coating, sputtering, plating, evaporation +<br>Vapor-assisted solution process (VASP) [51]<br>Vapor solid reaction (VSR)[52]<br>CVD [53]<br>Dipping [54]

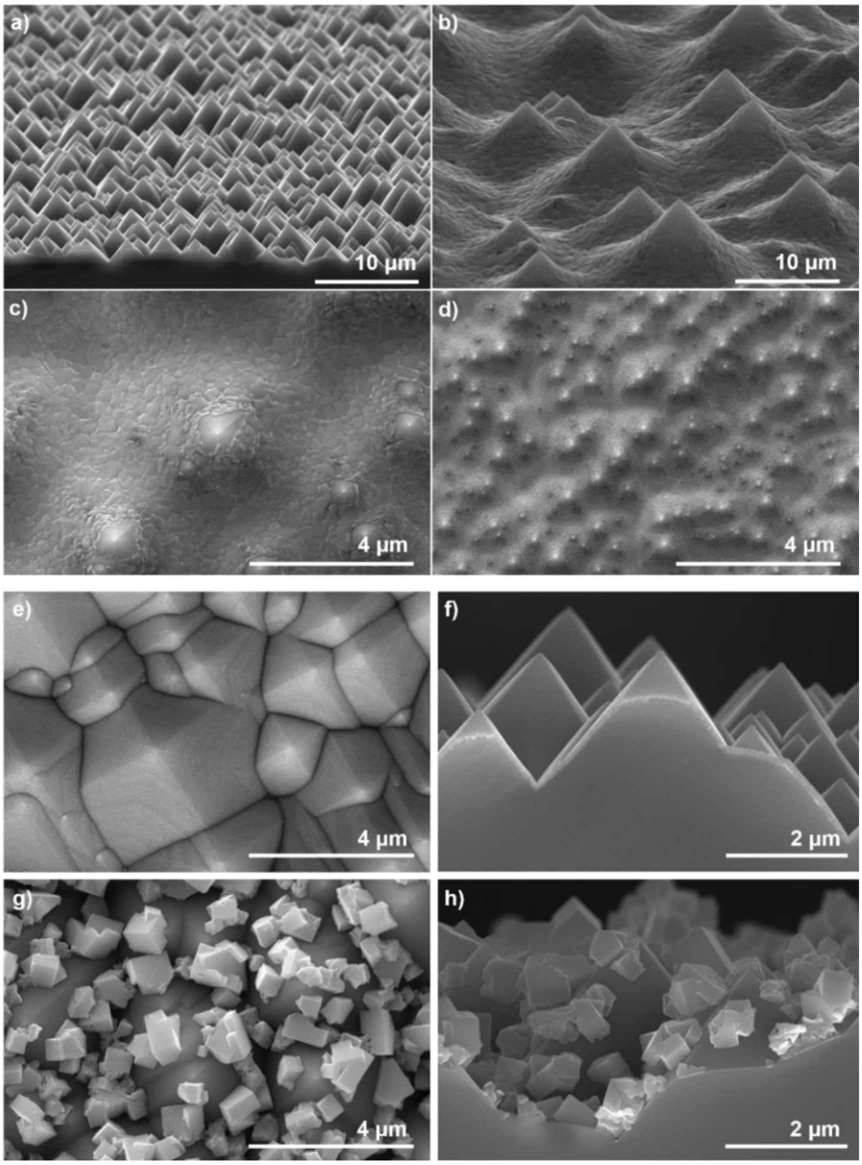

Supplementary Fig. 1. SEM images of perovskite on a randomly textured silicon surface fabricated by one-step spin coating and two-step dipping. It can be seen that the tips are not covered by perovskite. (**a**) Tilted view of the conventional silicon random texture, (**b**) spin-coated perovskite formed on the random texture, and (**c**),(**d**) top view of perovskite on the randomly textured silicon. Conformal PbO and perovskite fabricated by sputtering and dipping respectively. (**e**) and (**f**) are the top-view and cross-sectional images of PbO on the randomly textured silicon surface, and (**g**) and **h**) are the top-view and cross-sectional view of perovskite produced by immersion.

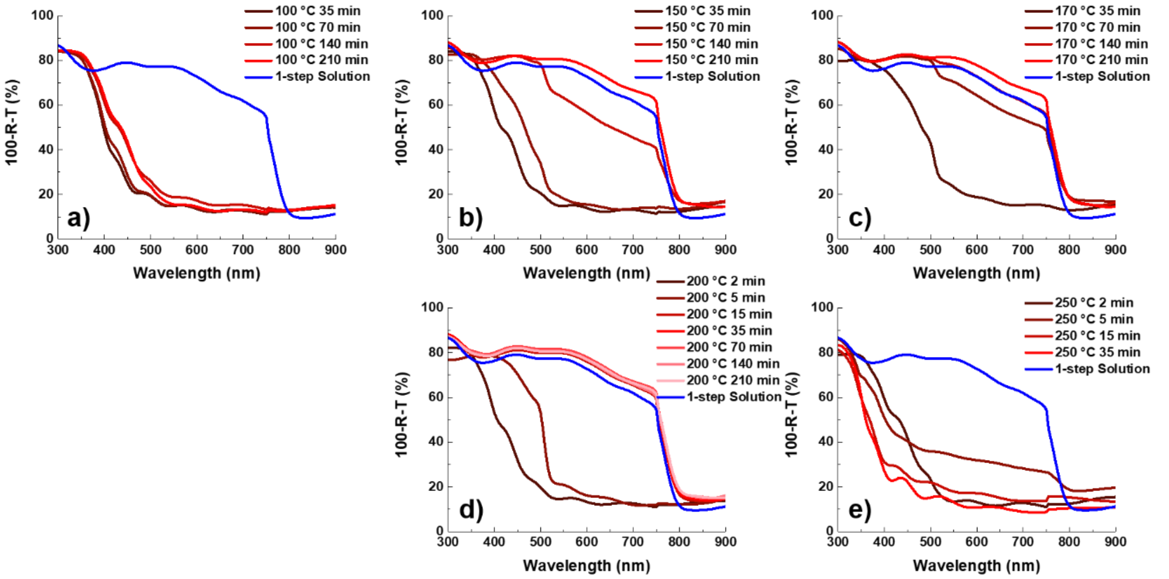

Supplementary Fig. 2. Light absorptance from 300 nm to 900 nm of films produced at **(a)** 100 °C, **(b)** 150 °C, **(c)** 170 °C, **(d)** 200 °C, and **(e)** 250 °C with corresponding conversion time. The conversion time is shown in the legend of each graph.

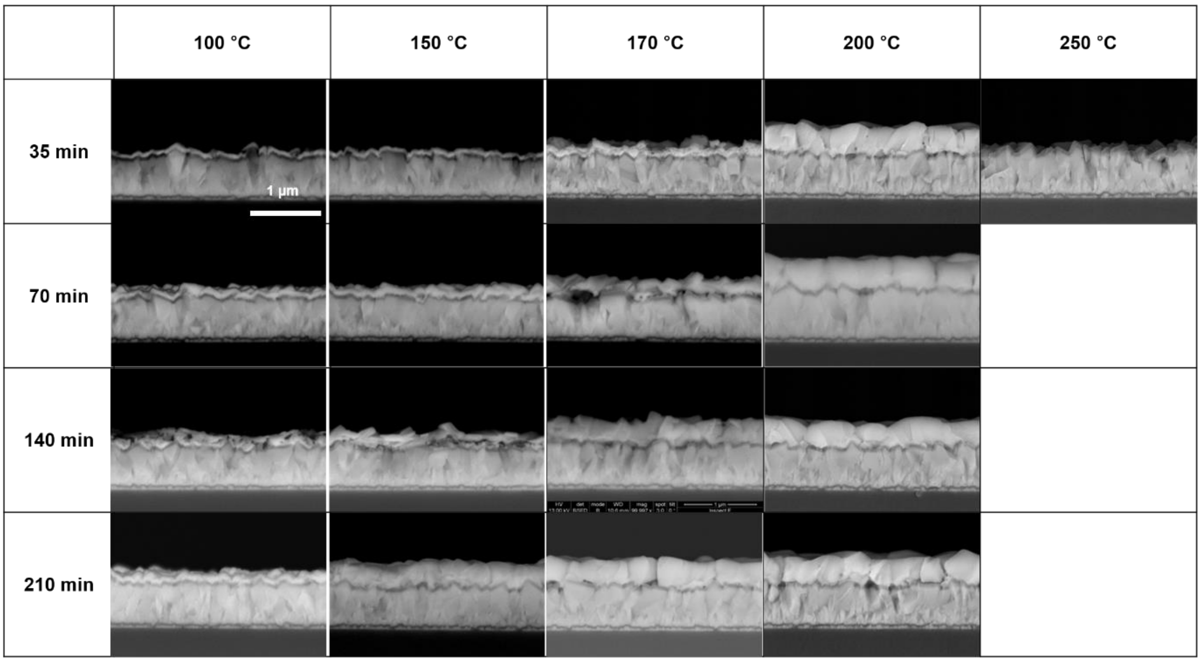

Supplementary Fig. 3. SEM cross section image of produced films depend on process temperature and time**.**

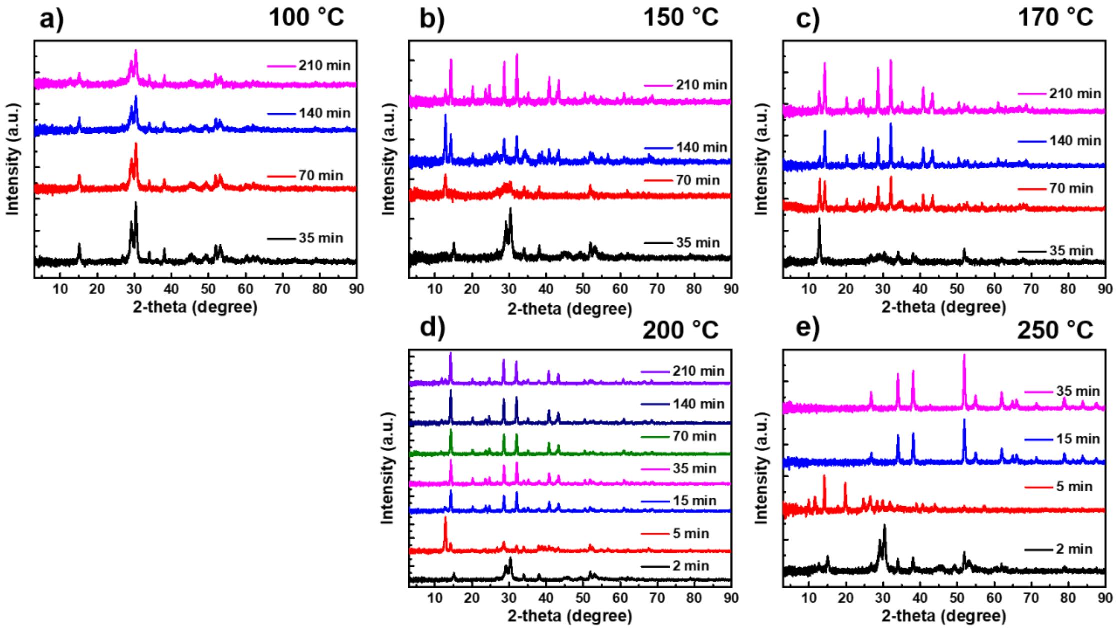

Supplementary Fig. 4. XRD peak intenstiy of films which produced by corresponding conversion temperature and time. **a)** 100 °C, **b)** 150 °C, **c)** 170 °C, **d)** 200 °C, and **e)** 250 °C

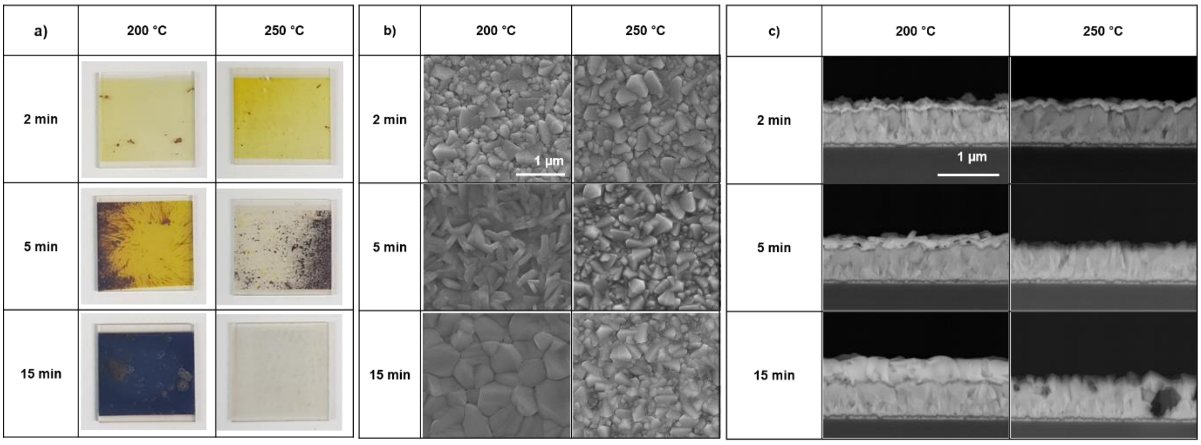

Supplementary Fig. 5. Image of films which fabricated with 2, 5, and 15 minute conversions at 200 and 250 °C. **a)** Digital camera image, **b)** SEM top view, **c)** SEM cross section view.

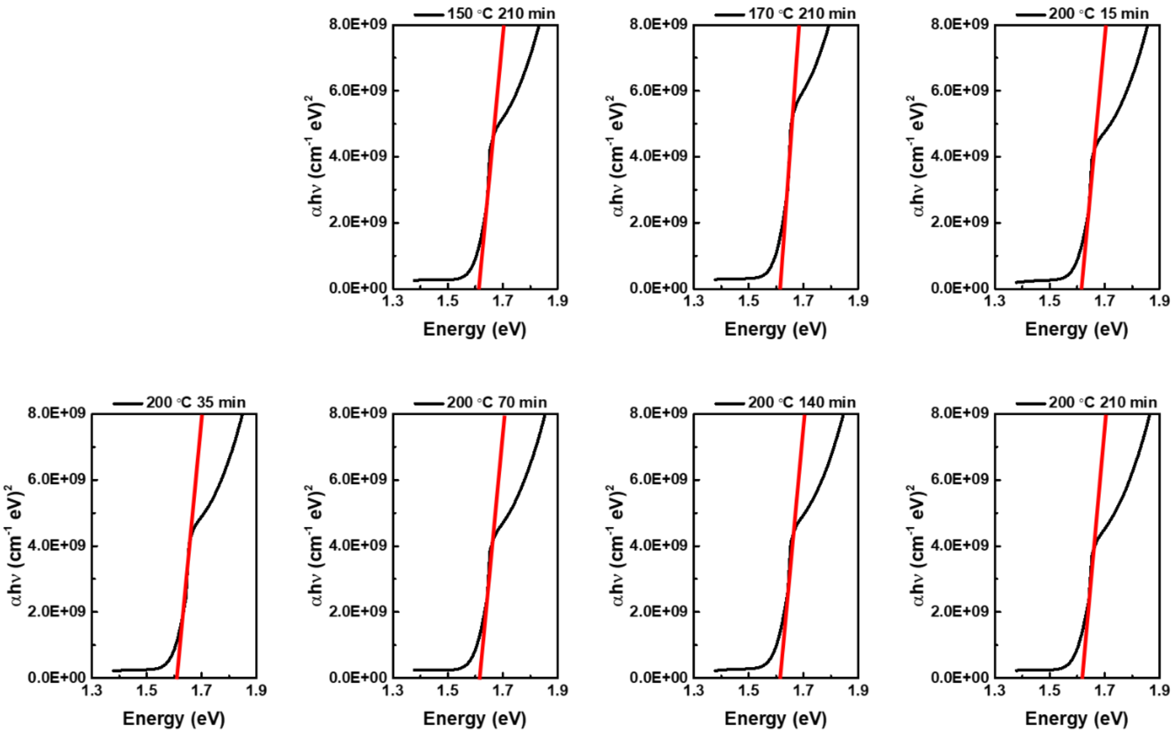

Supplementary Fig. 6. Tauc plot results of perovskite thin films fabricated at different temperatures and reaction times. The optical band gap was calculated between 1.60 eV and 1.62 eV [55].

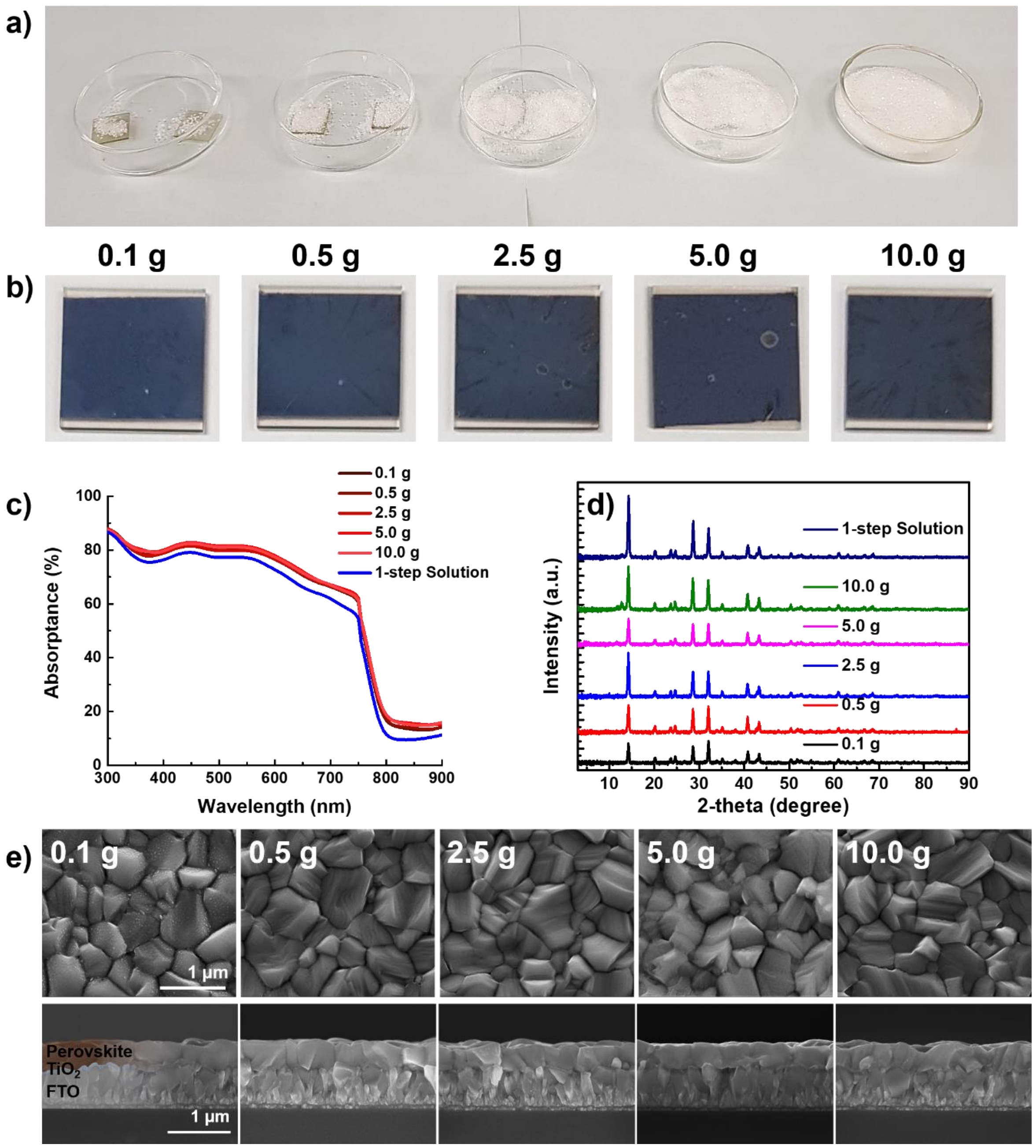

Supplementary Fig. 7. Results of 70 minute conversion process at 200 ° C depending on the amount of MAI. **a)** Digital camera image of different amount of MAI on PbO / TiO_2 / FTO film. **b)** perovskite film digital camera image after conversion process, **c)** light absorptance, **d)** XRD peaks, and e) SEM images. Inset colors in e) indicates guide to the eye.

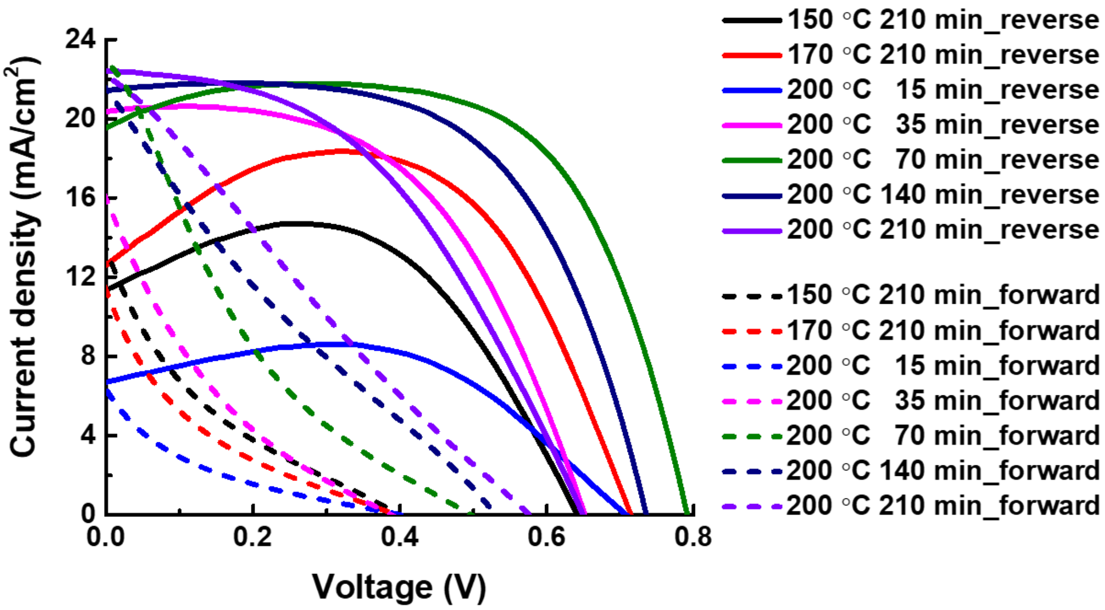

Supplementary Fig. 8. LIV measurement result of PbO converted perovskite solar cells produced at different conversion conditions. A hysteresis behavior can be originated from excessive ions, existing as interstitial defects, generated during dry two-step solid to solid conversion process [56]. In continuous work, the reason for the hysteresis behavior in our perovskite films should be investigated further.

Supplementary Table. 3. Solar cell parameters with different conversion conditions.

Reverse	V_{oc} (V)	J_{sc} (mA/cm^2)	Fill Factor (%)	R_{sh} (ohms)	R_s (ohms)	PCE (%)
150 °C 210 min	0.642	11.4	72.6	-800	178	5.3
170 °C 210 min	0.714	12.6	87.1	-503	138	7.9
200 °C 15 min	0.708	6.7	71.8	-1570	401	3.4
200 °C 35 min	0.652	20.4	53.5	-2460	122	7.1
200 °C 70 min	0.791	19.5	71.2	-699	79.8	11.1
200 °C 140 min	0.735	21.4	60.3	-3000	82.8	9.5
200 °C 210 min	0.649	22.4	45.1	6450	157	6.6
Forward						
150 °C 210 min	0.399	13.4	14.3	139	610	0.8
170 °C 210 min	0.394	11.3	12.7	122	937	0.6
200 °C 15 min	0.402	6.3	12.5	187	1870	0.3
200 °C 35 min	0.388	15.9	14.8	154	699	0.9
200 °C 70 min	0.496	22.9	15.2	330	621	1.7
200 °C 140 min	0.530	21.5	21.2	300	283	2.4
200 °C 210 min	0.576	22.2	23.9	573	381	3.1

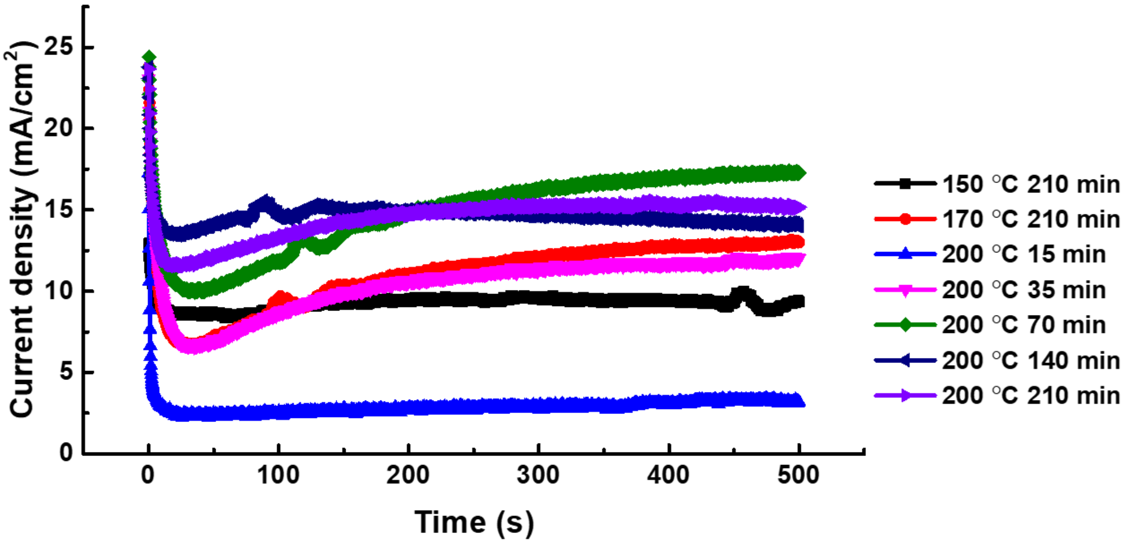

Supplementary Fig. 9. The 500 seconds current tracking result of PbO converted perovskite solar cells produced at different conversion conditions.

Supplementary Table. 4. Current density before and after 500 seconds current tracking under the AM1.5G condition.

	Pristine current density (mA/cm^2)	500 seconds after current density (mA/cm^2)
150 °C 210 min	11.4	9.3
170 °C 210 min	12.7	13.0
200 °C 15 min	6.7	3.2
200 °C 35 min	18.4	12.0
200 °C 70 min	19.5	17.2
200 °C 140 min	21.4	14.0
200 °C 210 min	21.0	15.2

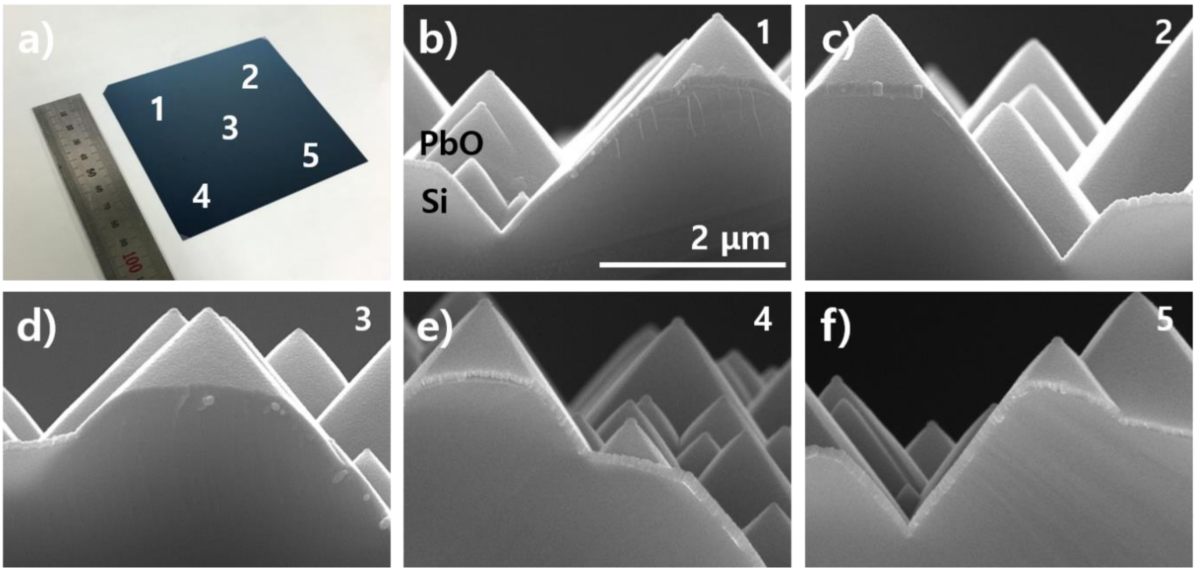

Supplementary Fig. 10. Conformal and uniform PbO precursor layer on a 4-inch randomly textured silicon surface. (**a**) Digital camera image of PbO on the 4-inch silicon surface and (**b**)–(**f**) cross-sectional SEM images of PbO on the textured silicon surface.

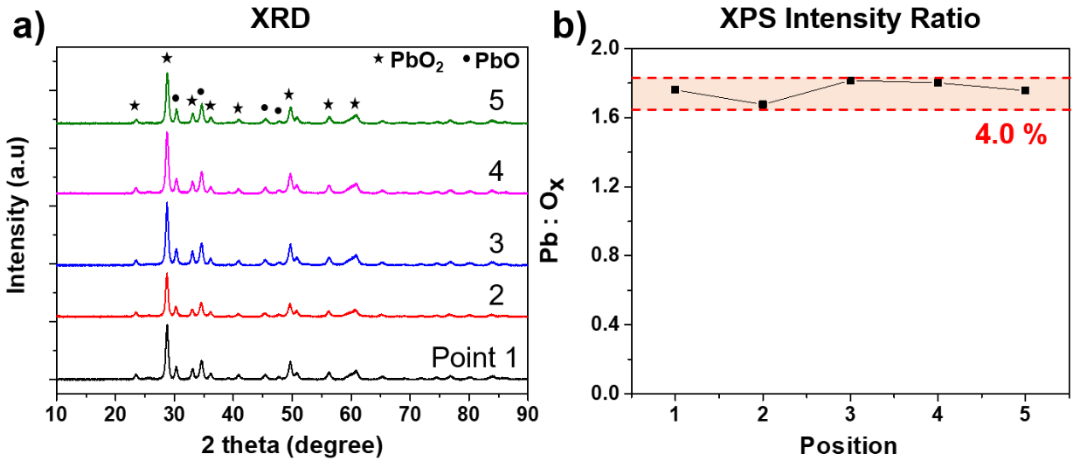

Supplementary Fig. 11 Uniformity demonstration of PbO_x precursor layer on 4-inch glass. (**a**) XRD peak position and (**b**) XPS peak intensity ratio between Pb and O.

Supplementary Table 5. Confirmed thickness and uniformity of conformal PbO on the 4-inch area. The thickness of the layer shown in this table obtain from Supplementary Fi g. 10 3-point averaged data with randomly selected points. Uniformity is calculated by (max-min)/2*average*100(%).

Point	1	2	3	4	5	Average	Uniformity
3-point average thickness [nm]	96	113	98	96	108	102	8.3%

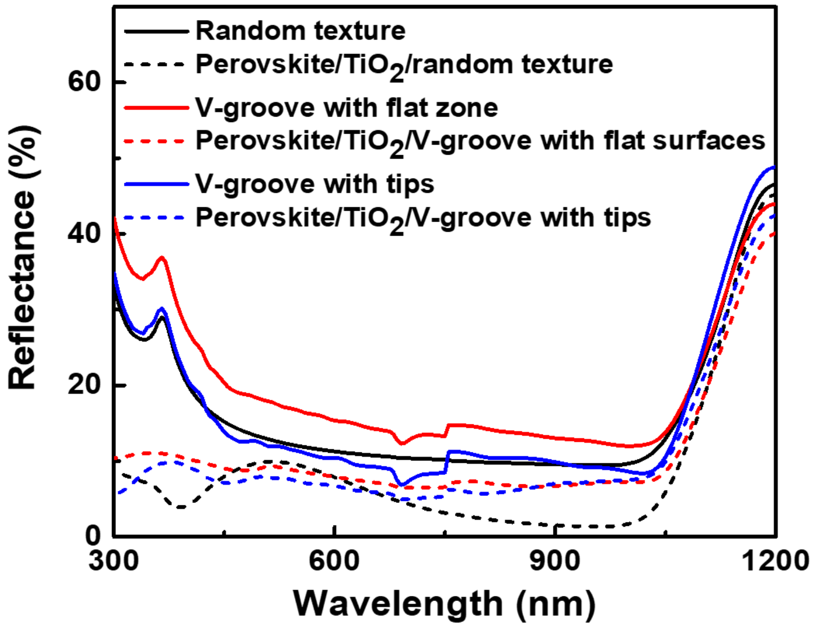

Supplementary Fig. 12. Optical reflectance obtained with different types of substrates.

Supplementary Table 6. Weighted reflectance of each device in the range from 300 nm to 1200 nm. Solar weighted reflectance (SWR) was calculated based on the supplementary equation 1 [57].

Device	Weighted reflectance (%)
Random texture	14.1
V-groove with flat zone	17.5
V-groove with tips	13.7
Random texture/TiO_2/perovskite	7.8
V-groove with flat zone/TiO_2/perovskite	9.9
V-groove with tips/TiO_2/perovskite	9.5

$$\textit{Solar Weighted Reflectance (SWR)} = \frac{\int_{300\,nm}^{1200\,nm} R(\lambda)Nphoton(\lambda)d\lambda}{\int_{300\,nm}^{1200\,nm} Nphoton(\lambda)d\lambda}$$

$R(\lambda)$: spectral reflectivity

$Nphoton$:photon number of the solar irradiation (AM1.5G) per unit area per unit wavelength.

Supplementary Equation 1. Equation for solar weighted reflectance calculation.

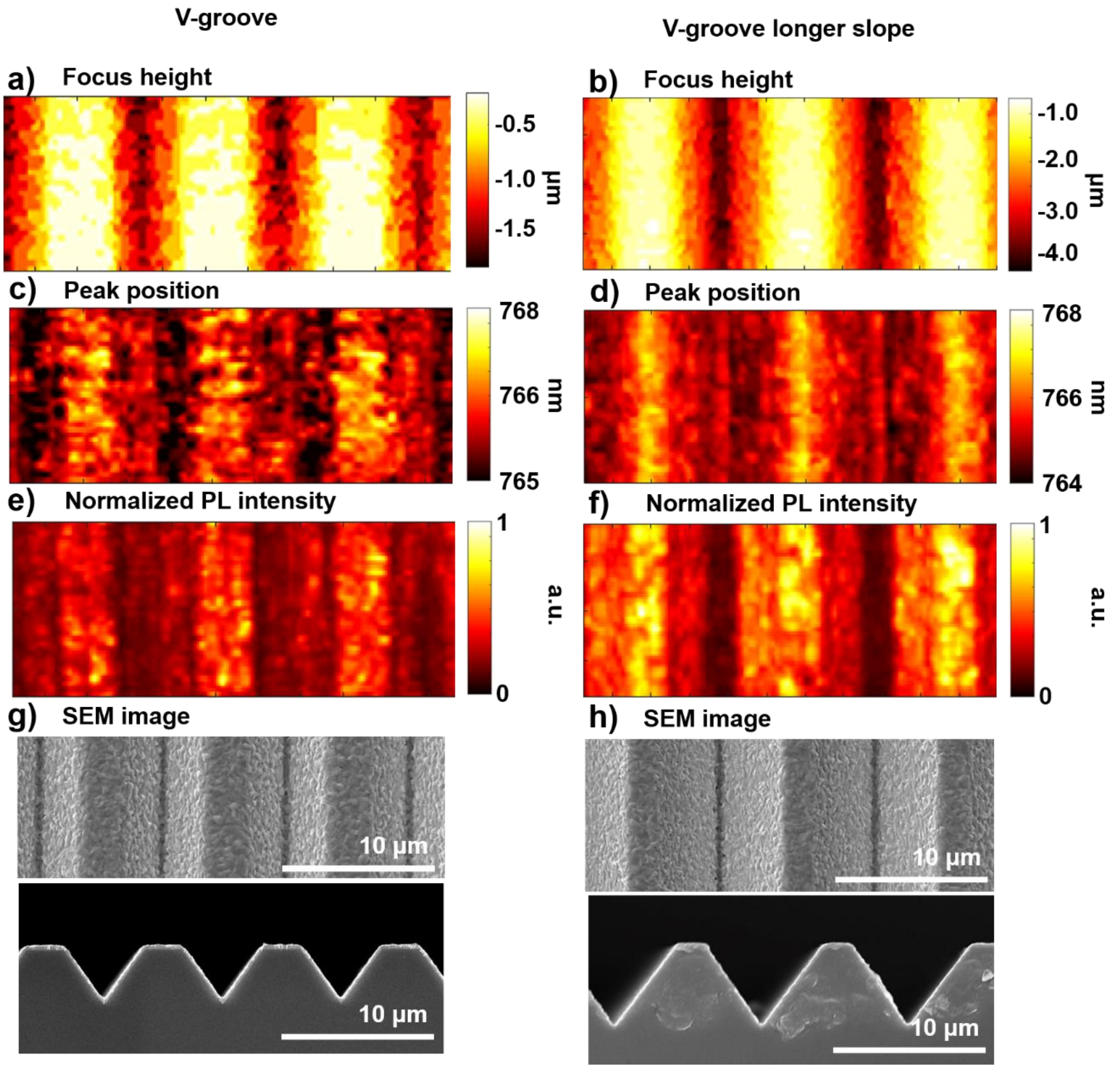

Supplementary Fig. 13. Combined image of the collective 2D maps for v-groove texture and v-groove texture with longer slope. **a),b)** Focus height, **c),d)** PL peak position, **e),f)** normalized PL intensity, and **g),h)** corresponding SEM image.

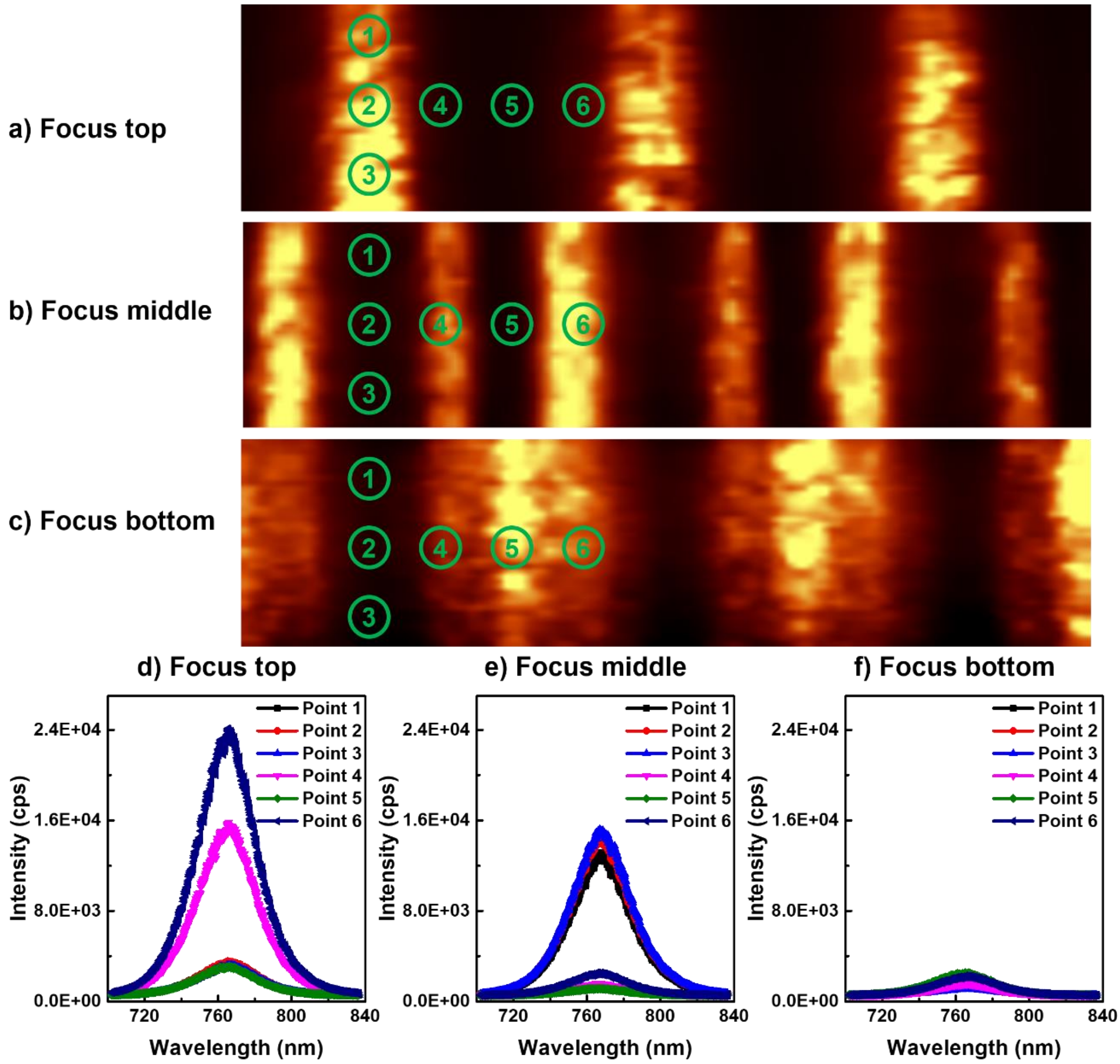

Supplementary Fig. 14. The results of displaying the PL spectrum at a specific position for specific focus heights. PL intensity obtained from **(a),(d)** focusing at top, **(b),(e)** focusing at middle, and **(c),(f)** focusing at bottom. It shows why 3D scanning is important when analyze conformal perovskite film on textured silicon surface. To analyze perovskite at specific position on textured surface with high XYZ resolutions, 2D PL intensity and peak position have to be collected throughout whole silicon texture thickness with different focus height and all the data should be combined into a comprehensive sinlge 2D image. Fig. 7 in the main manuscript summarize these process and shows combined images.

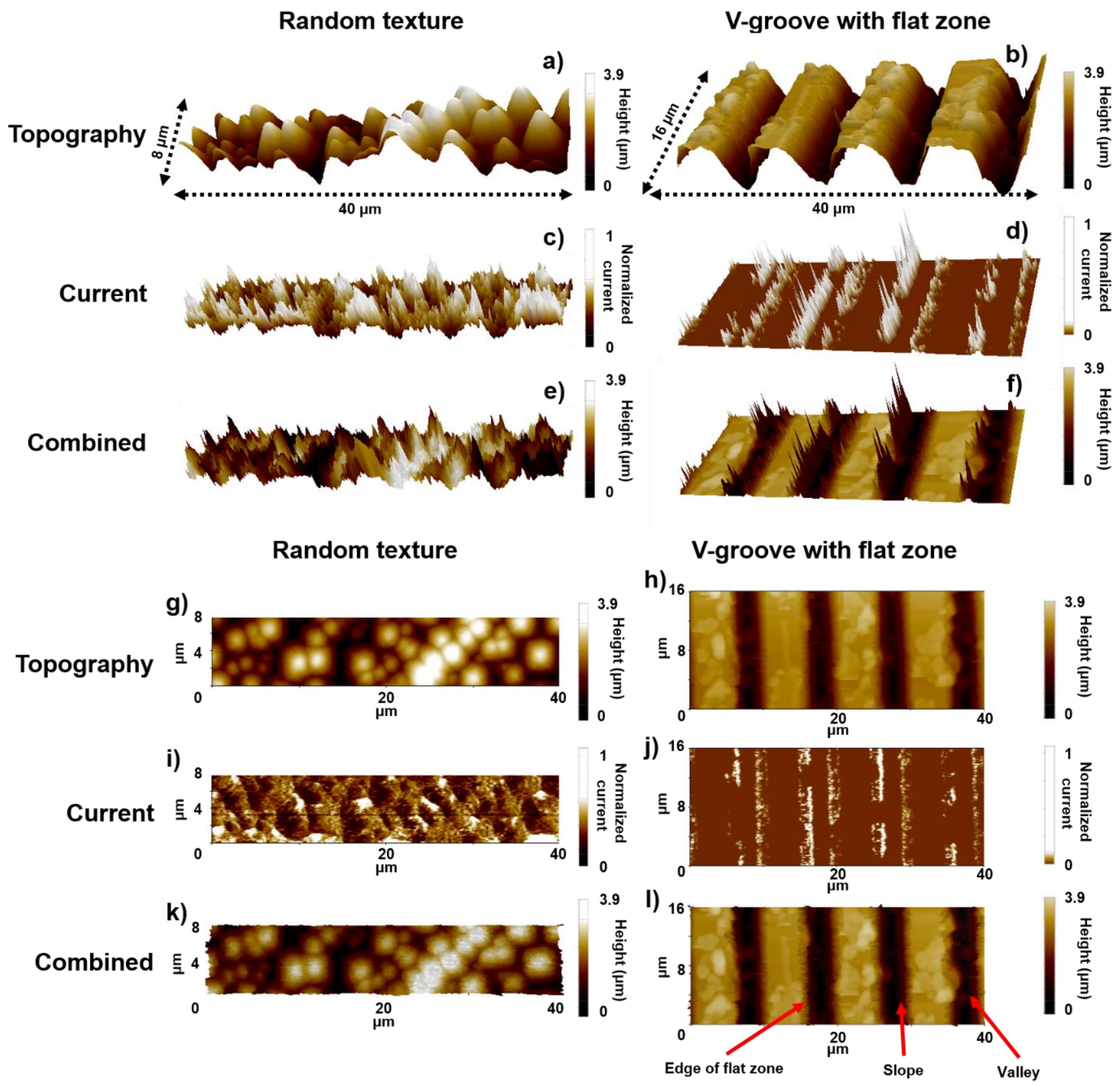

Supplementary Fig. 15. Angled 3D views of c-AFM measurement results. **a**),**b**) Topography, **c**),**d**) leakage current mapping. **e**),**f**) Combined images of topography and leakage current mapping. 2D views of c-AFM measurement results. (**g**) and (**h**) show the topography, (**i**) and (**j**) show the leakage current mapping, and (**k**) and (**l**) show the combined images of topography and leakage current mapping. The arrows in (**l**) indicate device positions. With respect to the perovskite solar cells on random texture and V-groove substrates, areas corresponding to 8 μm × 40 μm and 16 μm × 40 μm, respectively, are scanned. A bias of 1 V is applied between the samples and AFM tip.

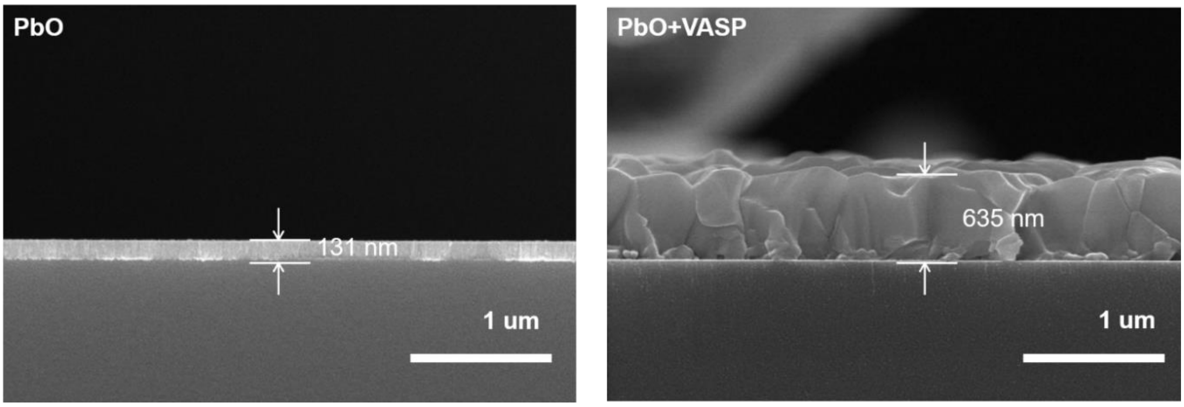

Supplementary Fig. 16. Volume expansion during the PbO-to-perovskite conversion process. Approximately 130 nm of PbO precursor film thickness was increased to approximately 630 nm of $CH_3NH_3PbI_3$ film.

Supplementary Table 7. Material parameters adopted from extant studies[58, 59]. These parameters were used for thermal simulation.

Material	Young's modulus [GPa]	Poisson's ratio
**Perovskite ($MAPbI_3$)**	12.8	0.33
**Si**	130	0.28

$$\frac{\partial \sigma_{xx}}{\partial x} + \frac{\partial \sigma_{xy}}{\partial y} = 0 \qquad \frac{\partial \sigma_{yy}}{\partial y} + \frac{\partial \sigma_{xy}}{\partial x} = 0$$

$$\sigma_{xx} + \sigma_{yy} = \frac{E}{(1+\nu)(1-2\nu)}\left(\frac{\partial u_x}{\partial x} + \frac{\partial u_y}{\partial y} - 2(1+\nu)\int_{T_1}^{T_2} LCTE\, dt\right) + 2\sigma^{int}$$

$$\sigma_{xx} - \sigma_{yy} = \frac{E}{(1+\nu)}\left(\frac{\partial u_x}{\partial x} - \frac{\partial u_y}{\partial y}\right)$$

$$\sigma_{xy} = \frac{E}{2(1+\nu)}\left(\frac{\partial u_x}{\partial y} + \frac{\partial u_y}{\partial x}\right)$$

E: Young's modulus

ν: Poisson's ratio

u_x, u_y: *Displacements in the x and y directions*

$LCTE$: *Linear coefficient for thermal expansion*

σ^{int}: Initial intrinsic stress in a deposited material layer

Supplementary Equation 2. Equations to calculate stress on isotropic homogeneous media by finite element analysis. Stresses in the *x* direction in the *x* plane and *y* direction in the *y* plane are calculated and expressed as a contour map, which is shown in Fig. 9 in the main manuscript.

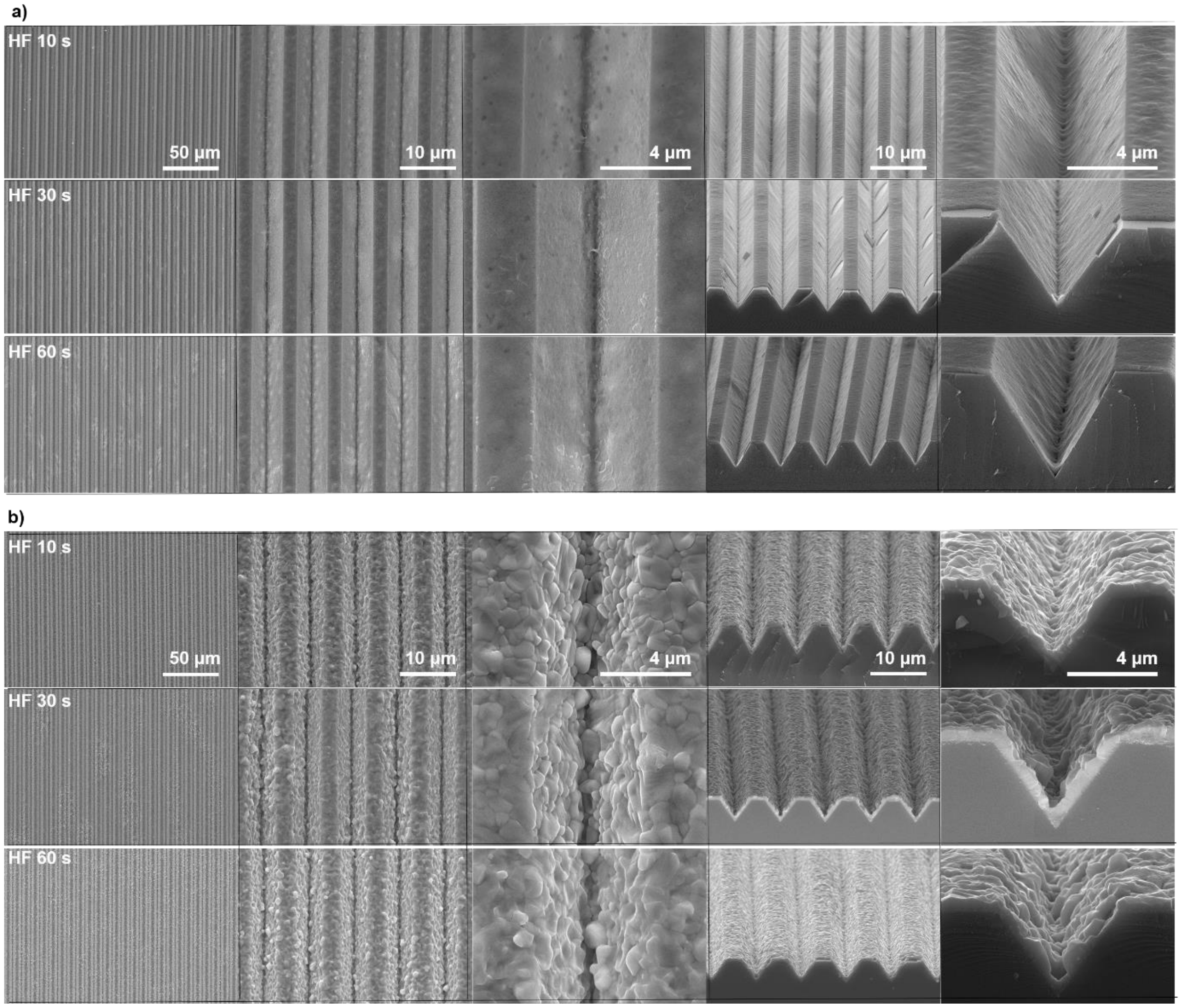

Supplementary Fig. 17. a) PbO precursors with 10, 30, and 60 s HF treatments and **b**) the corresponding converted perovskite layers on a V-groove texture.

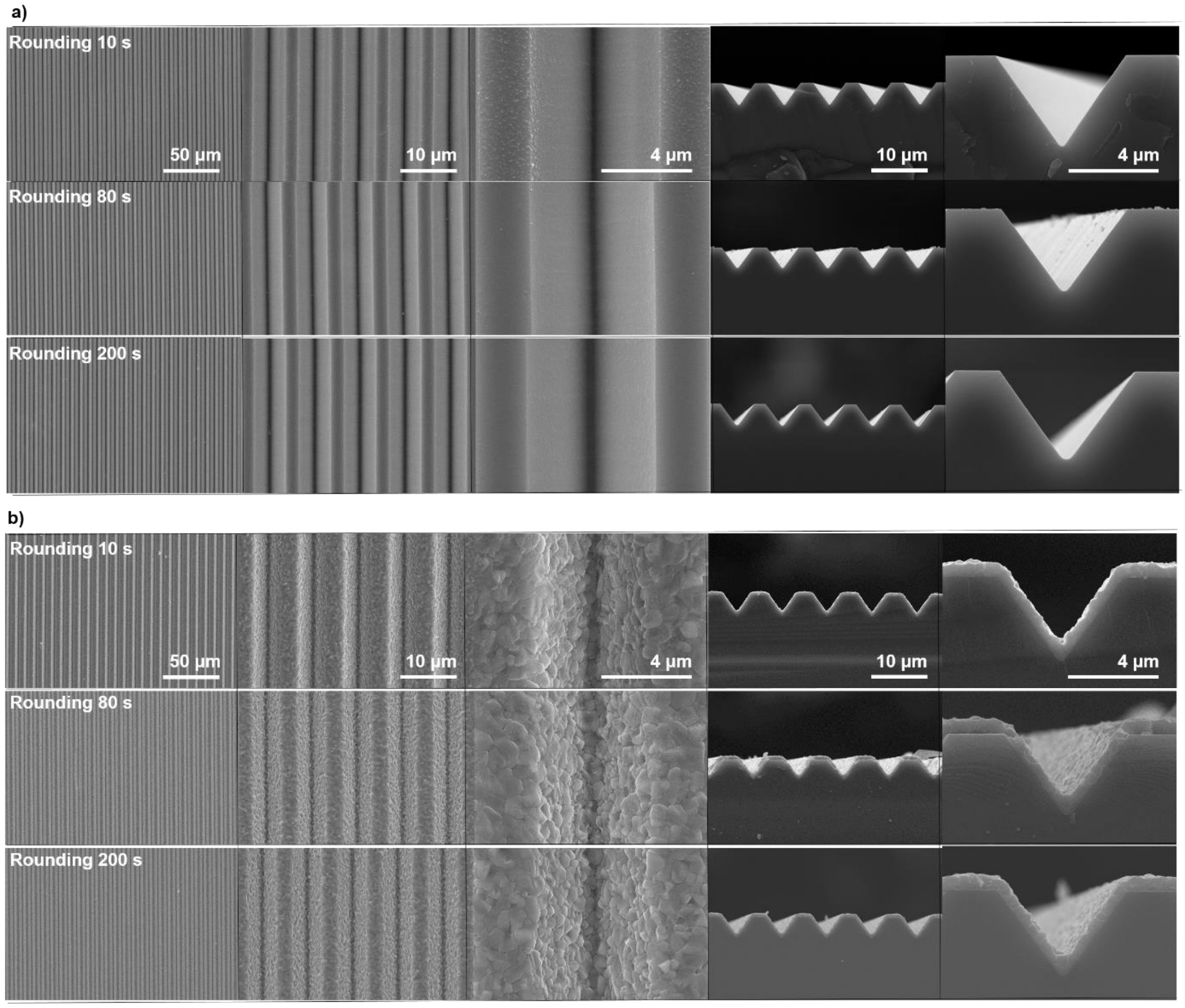

Supplementary Fig. 18. a) PbO precursors on 10, 80, and 200 s chemically rounded V-groove textures and **b**) the converted perovskite layers on the corresponding substrates.

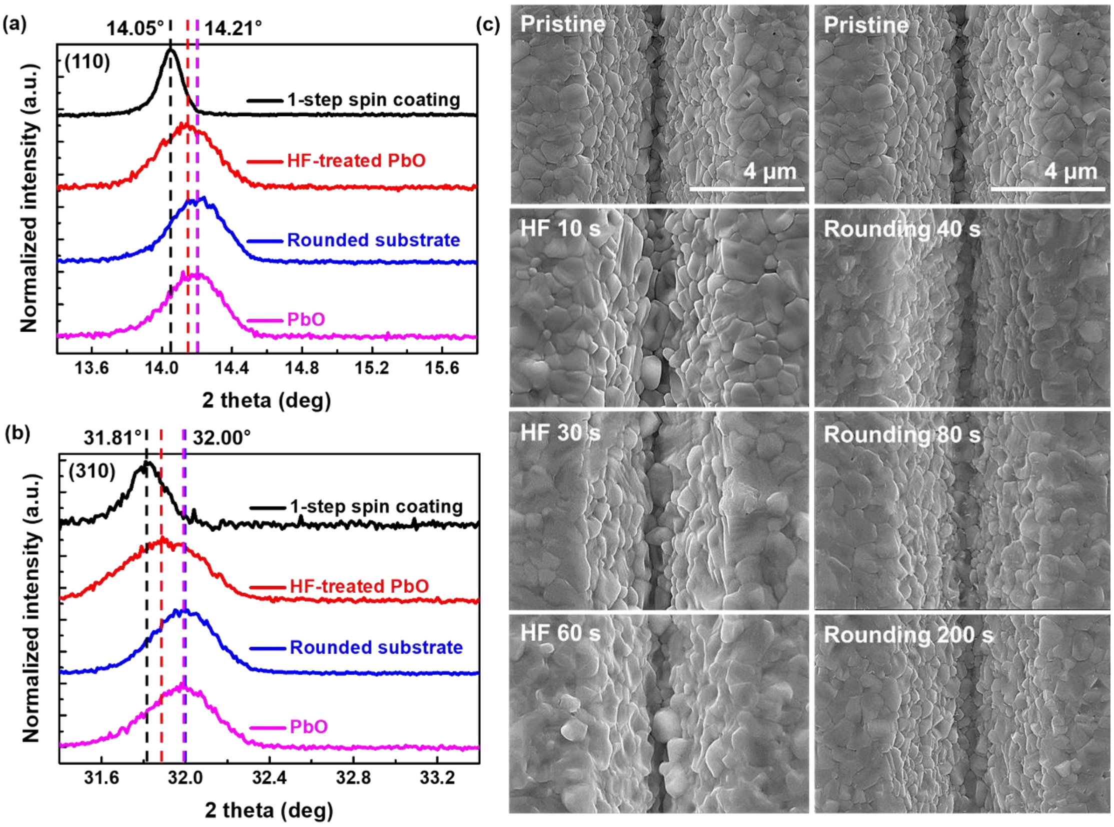

Supplementary Fig. 19. Normalized XRD data at the **a)** (110) and **b)** (310) planes. Top line: 1-step spin-coated perovskite, second line: converted perovskite from HF 10 s treated PbO, third line: converted perovskite on 200 s chemically rounded substrate, fourth line: converted perovskite from pristine PbO. **c)** SEM images of converted perovskite from differently HF -treated precursors and perovskite on differently rounded substrates.

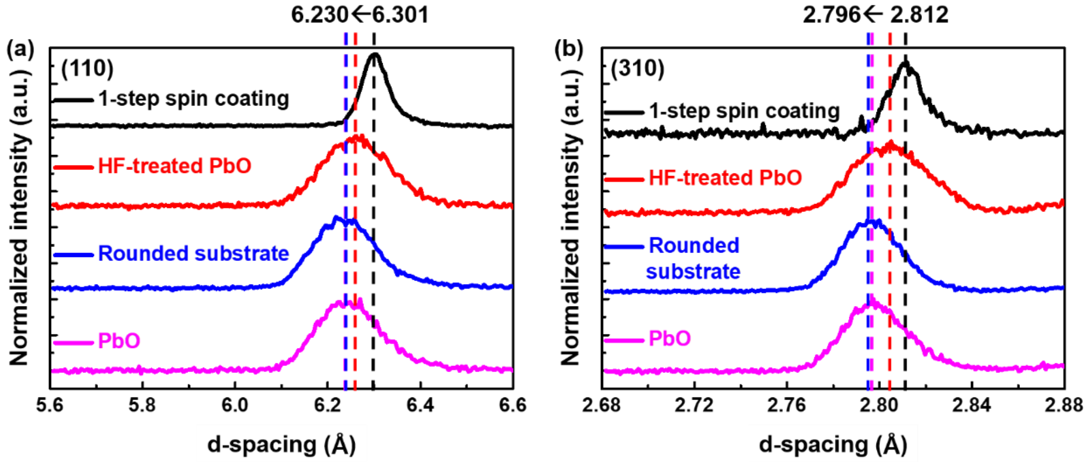

Supplementary Fig. 20. The d-spacing shifts calculated from the XRD data shown in Fig. 10 at the **(a)** (110) and **(b)** (310) planes.

Supplementary Table 8. XRD peak position and d-spacing calculations for the perovskite layer.

(110)	1-step spin coating	HF-treated PbO	PbO	Rounded substrate
**2 theta**	14.05	14.16	14.20	14.21
**d spacing [Å]**	6.301	6.252	6.235	6.230
**Difference [Å]**	-	0.049	0.066	0.071
**Strain**	-	0.0078	0.0106	0.0113
**Stress [MPa]**	-	77.9 – 155.8	106.2 – 212.4	113.3 – 226.6
**(310)**				
**2 theta**	31.81	31.92	31.99	32.00
**d spacing [Å]**	2.812	2.803	2.797	2.796
**Difference [Å]**	-	0.009	0.015	0.016
**Strain**	-	0.0034	0.0055	0.0058
**Stress [MPa]**	-	33.7 – 67.4	55.1 – 110.2	58.2 – 116.3

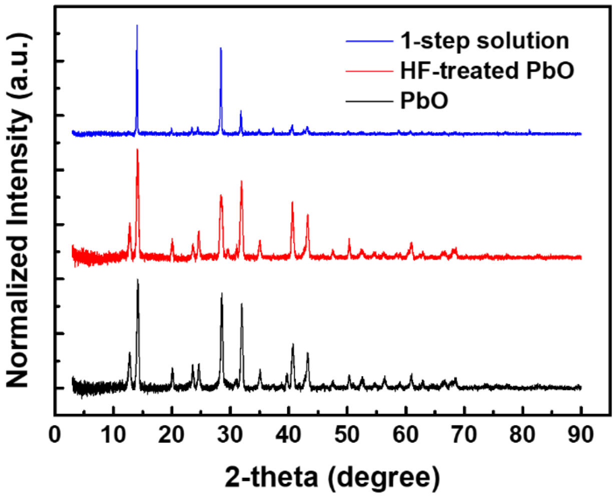

Supplementary Fig. 21. The XRD of the total angle was compared with the perovskite prepared by the 1-step solution method, perovskite prepared with PbO, and HF-treated PbO.

Supplementary References

1. Mailoa JP*, et al.* A 2-terminal perovskite/silicon multijunction solar cell enabled by a silicon tunnel junction. *Applied Physics Letters* **106**, 121105 (2015).

2. Albrecht S*, et al.* Monolithic perovskite/silicon-heterojunction tandem solar cells processed at low temperature. *Energy & Environmental Science* **9**, 81-88 (2016).

3. Werner J*, et al.* Efficient monolithic perovskite/silicon tandem solar cell with cell area> 1 cm2. *The journal of physical chemistry letters* **7**, 161-166 (2015).

4. Werner Jrm*, et al.* Efficient near-infrared-transparent perovskite solar cells enabling direct comparison of 4-terminal and monolithic perovskite/silicon tandem cells. *Acs Energy Letters* **1**, 474-480 (2016).

5. Werner J*, et al.* Zinc tin oxide as high-temperature stable recombination layer for mesoscopic perovskite/silicon monolithic tandem solar cells. *Applied Physics Letters* **109**, (2016).

6. Bush KA*, et al.* 23.6%-efficient monolithic perovskite/silicon tandem solar cells with improved stability. *Nature Energy* **2**, 17009 (2017).

7. Sahli F*, et al.* Improved optics in monolithic perovskite/silicon tandem solar cells with a nanocrystalline silicon recombination junction. *Advanced Energy Materials* **8**, 1701609 (2018).

8. Wu Y*, et al.* Monolithic perovskite/silicon-homojunction tandem solar cell with over 22% efficiency. *Energy & Environmental Science* **10**, 2472-2479 (2017).

9. Fan R*, et al.* Toward full solution processed perovskite/Si monolithic tandem solar device with PCE exceeding 20%. *Solar RRL* **1**, 1700149 (2017).

10. Zhu S*, et al.* Transparent electrode for monolithic perovskite/silicon-heterojunction two-terminal tandem solar cells. *Nano Energy* **45**, 280-286 (2018).

11. Sahli F*, et al.* Fully textured monolithic perovskite/silicon tandem solar cells with 25.2% power conversion efficiency. *Nature materials*, 1 (2018).

12. Green MA, Hishikawa Y, Dunlop ED, Levi DH, Hohl-Ebinger J, Ho-Baillie AWY. Solar cell efficiency tables (version 52). *Prog Photovoltaics* **26**, 427-436 (2018).

13. Oxford PV Sets World Record for Perovskite Solar Cell. (2018). Available at: https://www.oxfordpv.com/news/oxford-pv-sets-world-record-perovskite-solar-cell (2018).

14. Zheng J*, et al.* Large area efficient interface layer free monolithic perovskite/homo-junction-silicon tandem solar cell with over 20% efficiency. *Energy & Environmental Science*, (2018).

15. Zheng J*, et al.* 21.8% efficient monolithic perovskite/homo-junction-silicon tandem solar cell on 16 cm2. *ACS Energy Letters* **3**, 2299-2300 (2018).

16. Bush KA*, et al.* Minimizing current and voltage losses to reach 25% efficient monolithic two-terminal perovskite–silicon tandem solar cells. *ACS Energy Letters* **3**, 2173-2180 (2018).

17. Chen B*, et al.* Grain Engineering for Perovskite/Silicon Monolithic Tandem Solar Cells

with Efficiency of 25.4%. *Joule*, (2018).

18. Jošt M, *et al.* Textured interfaces in monolithic perovskite/silicon tandem solar cells: Advanced light management for improved efficiency and energy yield. *Energy & Environmental Science*, (2019).

19. Qiu Z, *et al.* Monolithic perovskite/Si tandem solar cells exceeding 22% efficiency via optimizing top cell absorber. *Nano energy* **53**, 798-807 (2018).

20. Zhu S, *et al.* Solvent Engineering to Balance Light Absorbance and Transmittance in Perovskite for Tandem Solar Cells. *Solar RRL* **2**, 1800176 (2018).

21. Hou F, *et al.* Inverted pyramidally-textured PDMS antireflective foils for perovskite/silicon tandem solar cells with flat top cell. *Nano energy* **56**, 234-240 (2019).

22. Green MA, Dunlop ED, Levi DH, Hohl-Ebinger J, Yoshita M, Ho-Baillie AWY. Solar cell efficiency tables (version 54). *Prog Photovoltaics* **27**, 565-575 (2019).

23. Shen H, *et al.* In situ recombination junction between p-Si and TiO2 enables high-efficiency monolithic perovskite/Si tandem cells. *Science advances* **4**, eaau9711 (2018).

24. Park IJ, Park JH, Ji SG, Park M-A, Jang JH, Kim JY. A Three-Terminal Monolithic Perovskite/Si Tandem Solar Cell Characterization Platform. *Joule* **3**, 807-818 (2019).

25. Hou F, *et al.* Monolithic Perovskite/Silicon-Heterojunction Tandem Solar Cells with Open-Circuit Voltage of over 1.8 V. *ACS Applied Energy Materials* **2**, 243-249 (2019).

26. Mazzarella L, *et al.* Infrared Light Management Using a Nanocrystalline Silicon Oxide Interlayer in Monolithic Perovskite/Silicon Heterojunction Tandem Solar Cells with Efficiency above 25%. *Advanced Energy Materials*, 1803241 (2019).

27. Nogay G, *et al.* 25.1%-Efficient Monolithic Perovskite/Silicon Tandem Solar Cell Based on ap-type Monocrystalline Textured Silicon Wafer and High-Temperature Passivating Contacts. *ACS Energy Letters* **4**, 844-845 (2019).

28. Kim CU, *et al.* Optimization of device design for low cost and high efficiency planar monolithic perovskite/silicon tandem solar cells. *Nano Energy*, (2019).

29. Kamino BA, *et al.* Low-Temperature Screen-Printed Metallization for the Scale-Up of Two-Terminal Perovskite–Silicon Tandems. *ACS Applied Energy Materials*, (2019).

30. Köhnen E, *et al.* Highly efficient monolithic perovskite silicon tandem solar cells: analyzing the influence of current mismatch on device performance. *Sustainable Energy & Fuels*, (2019).

31. Ramírez Quiroz CO, *et al.* Interface Molecular Engineering for Laminated Monolithic Perovskite/Silicon Tandem Solar Cells with 80.4% Fill Factor. *Advanced Functional Materials*, 1901476 (2019).

32. Choi IY, *et al.* Two-terminal mechanical perovskite/silicon tandem solar cells with transparent conductive adhesives. *Nano Energy* **65**, 104044 (2019).

33. Zheng J, *et al.* Large Area 23%-Efficient Monolithic Perovskite/Homo-Junction-Silicon Tandem Solar Cell With Enhanced UV Stability Using Down-Shifting Material. *ACS Energy Letters*, (2019).

34. Werner J*, et al.* Perovskite/perovskite/silicon monolithic triple-junction solar cells with a fully textured design. *ACS Energy Letters* **3**, 2052-2058 (2018).

35. Ahn N, Son D-Y, Jang I-H, Kang SM, Choi M, Park N-G. Highly reproducible perovskite solar cells with average efficiency of 18.3% and best efficiency of 19.7% fabricated via Lewis base adduct of lead (II) iodide. *J Am Chem Soc* **137**, 8696-8699 (2015).

36. Jeon NJ, Noh JH, Kim YC, Yang WS, Ryu S, Seok SI. Solvent engineering for high-performance inorganic–organic hybrid perovskite solar cells. *Nature materials* **13**, 897 (2014).

37. Priyadarshi A*, et al.* A large area (70 cm 2) monolithic perovskite solar module with a high efficiency and stability. *Energy & Environmental Science* **9**, 3687-3692 (2016).

38. Ye F*, et al.* Soft-cover deposition of scaling-up uniform perovskite thin films for high cost-performance solar cells. *Energy & Environmental Science* **9**, 2295-2301 (2016).

39. Razza S*, et al.* Perovskite solar cells and large area modules (100 cm2) based on an air flow-assisted PbI2 blade coating deposition process. *Journal of Power Sources* **277**, 286-291 (2015).

40. Deng Y, Peng E, Shao Y, Xiao Z, Dong Q, Huang J. Scalable fabrication of efficient organolead trihalide perovskite solar cells with doctor-bladed active layers. *Energy & Environmental Science* **8**, 1544-1550 (2015).

41. Cotella G*, et al.* One-step deposition by slot-die coating of mixed lead halide perovskite for photovoltaic applications. *Solar Energy Materials and Solar Cells* **159**, 362-369 (2017).

42. Razza S, Castro-Hermosa S, Di Carlo A, Brown TM. Research update: large-area deposition, coating, printing, and processing techniques for the upscaling of perovskite solar cell technology. *APL Materials* **4**, 091508 (2016).

43. Burschka J*, et al.* Sequential deposition as a route to high-performance perovskite-sensitized solar cells. *Nature* **499**, 316 (2013).

44. Xiao Z*, et al.* Efficient, high yield perovskite photovoltaic devices grown by interdiffusion of solution-processed precursor stacking layers. *Energy & Environmental Science* **7**, 2619-2623 (2014).

45. Momblona C*, et al.* Efficient vacuum deposited pin and nip perovskite solar cells employing doped charge transport layers. *Energy & Environmental Science* **9**, 3456-3463 (2016).

46. Gil-Escrig L, Momblona C, La-Placa MG, Boix PP, Sessolo M, Bolink HJ. Vacuum Deposited Triple-Cation Mixed-Halide Perovskite Solar Cells. *Advanced Energy Materials* **8**, 1703506 (2018).

47. Tavakoli MM*, et al.* Fabrication of efficient planar perovskite solar cells using a one-step chemical vapor deposition method. *Scientific reports* **5**, 14083 (2015).

48. Hsiao SY*, et al.* Efficient All-Vacuum Deposited Perovskite Solar Cells by Controlling Reagent Partial Pressure in High Vacuum. *Advanced Materials* **28**, 7013-7019 (2016).

49. Leyden MR, Ono LK, Raga SR, Kato Y, Wang S, Qi Y. High performance perovskite solar cells by hybrid chemical vapor deposition. *Journal of Materials Chemistry A* **2**, 18742-18745 (2014).

50. Raifuku I, Ishikawa Y, Bourgeteau T, Bonnassieux Y, i Cabarrocas PR, Uraoka Y. Fabrication of perovskite solar cells using sputter-processed CH3NH3PbI3 films. *Applied Physics Express* **10**, 094101 (2017).

51. Chen Q*, et al.* Planar heterojunction perovskite solar cells via vapor-assisted solution process. *J Am Chem Soc* **136**, 622-625 (2013).

52. Chen X*, et al.* Large-area, high-quality organic–inorganic hybrid perovskite thin films via a controlled vapor–solid reaction. *Journal of Materials Chemistry A* **4**, 9124-9132 (2016).

53. Li M-H, Shen P-S, Chen J-S, Chiang Y-H, Chen P, Guo T-F. Low-pressure hybrid chemical vapor deposition for efficient perovskite solar cells and module. In: *Active-Matrix Flatpanel Displays and Devices (AMFPD), 2016 The 23rd International Workshop on Active-Matrix Flatpanel Displays and Devices* (ed^(eds). IEEE (2016).

54. Zhang Z, Li M, Liu W, Yue X, Cui P, Wei D. CH3NH3PbI3 converted from reactive magnetron sputtered PbO for large area perovskite solar cells. *Solar Energy Materials and Solar Cells* **163**, 250-254 (2017).

55. Kim H-S*, et al.* Lead iodide perovskite sensitized all-solid-state submicron thin film mesoscopic solar cell with efficiency exceeding 9%. *Scientific reports* **2**, 591 (2012).

56. Snaith, H. J*, et al.* Anomalous hysteresis in perovskite solar cells. *The journal of physical chemistry letters* **5(9)**, 1511-1515 (2014).

57. Sai H, Kanamori Y, Arafune K, Ohshita Y, Yamaguchi M. Light trapping effect of submicron surface textures in crystalline Si solar cells. *Prog Photovoltaics* **15**, 415-423 (2007).

58. Feng J. Mechanical properties of hybrid organic-inorganic CH3NH3BX3 (B= Sn, Pb; X= Br, I) perovskites for solar cell absorbers. *Apl Materials* **2**, 081801 (2014).

59. Wortman J, Evans R. Young's modulus, shear modulus, and Poisson's ratio in silicon and germanium. *Journal of applied physics* **36**, 153-156 (1965).
