# Supplementary material for: Perovskites fabricated on textured silicon surfaces for tandem solar cells
Source: Commun Chem. 2020 Mar 25;3:37. doi: 10.1038/s42004-020-0283-4 (PMC9814648; doi:10.1038/s42004-020-0283-4)
Supplement: Supplementary file 3 — Reporting Summary [file 42004_2020_283_MOESM3_ESM.pdf]

nature research

Corresponding author(s): DBPR COMMSCHEM-19-0121-T

Solar Cells Reporting Summary

Nature Research wishes to improve the reproducibility of the work that we publish. This form is intended for publication with all accepted papers reporting the characterization of photovoltaic devices and provides structure for consistency and transparency in reporting. Some list items might not apply to an individual manuscript, but all fields must be completed for clarity.

For further information on Nature Research policies, including our data availability policy, see Authors & Referees.

ü Experimental design

Please check: are the following details reported in the manuscript?

1. Dimensions

Item	Yes	No	Details
Area of the tested solar cells	x		Each data point was adopted from the LIV reverse scan results and the measurements were conducted with 0.078 cm2 shadow mask under the AM1.5G condition
Method used to determine the device area	x		The area of the mask was measured by optical microscope and optical equipment at Korea Institute of Energy Research.

2. Current-voltage characterization

Item	Yes	No	Details
Current density-voltage (J-V) plots in both forward and backward direction	x		The LIV curves and the maximum power point tacking results for each solar cells are in the Supplementary Fig. 8,9 and Supplementary Table 3,4.
Voltage scan conditions<br>*For instance: scan direction, speed, dwell times*	x		The LIV curves and the maximum power point tacking results for each solar cells are in the Supplementary Fig. 8,9 and Supplementary Table 3,4.
Test environment<br>*For instance: characterization temperature, in air or in glove box*			*State where this information can be found in the text.*<br>*Explain why this information is not reported/not relevant.*
Protocol for preconditioning of the device before its characterization			*State where this information can be found in the text.*<br>*Explain why this information is not reported/not relevant.*
Stability of the J-V characteristic<br>*Verified with time evolution of the maximum power point or with the photocurrent at maximum power point; see ref. 7 for details.*	x		The LIV curves and the maximum power point tacking results for each solar cells are in the Supplementary Fig. 8,9 and Supplementary Table 3,4.

3. Hysteresis or any other unusual behaviour

Item	Yes	No	Details
Description of the unusual behaviour observed during the characterization	x		Hysteresis was large when measuring solar cell efficiency, and there were cases where FF was over estimated.
Related experimental data	x		Supplementary Fig. 8,9 and Supplementary Table 3,4.

4. Efficiency

Item	Yes	No	Details
External quantum efficiency (EQE) or incident photons to current efficiency (IPCE)		x	*Explain why this information is not reported/not relevant.*
A comparison between the integrated response under the standard reference spectrum and the response measure under the simulator			*State where this information can be found in the text.*<br>*Explain why this information is not reported/not relevant.*
For tandem solar cells, the bias illumination and bias voltage used for each subcell			*State where this information can be found in the text.*<br>*Explain why this information is not reported/not relevant.*

5. Calibration

Item	Yes	No	Details
Light source and reference cell or sensor used for the characterization			*State where this information can be found in the text.*<br>*Explain why this information is not reported/not relevant.*

Confirmation that the reference cell was calibrated and certified

- [ ] Yes — State where this information can be found in the text.
- [ ] No — Explain why this information is not reported/not relevant.

Calculation of spectral mismatch between the reference cell and the devices under test

- [ ] Yes — State where this information can be found in the text.
- [ ] No — Explain why this information is not reported/not relevant.

6. Mask/aperture

Size of the mask/aperture used during testing

- [x] Yes — Each data point was adopted from the LIV reverse scan results and the measurements were conducted with 0.078 cm2 shadow mask under the AM1.5G condition
- [ ] No

Variation of the measured short-circuit current density with the mask/aperture area

- [ ] Yes — State where this information can be found in the text.
- [ ] No — Explain why this information is not reported/not relevant.

7. Performance certification

Identity of the independent certification laboratory that confirmed the photovoltaic performance

- [ ] Yes
- [x] No — Explain why this information is not reported/not relevant.

A copy of any certificate(s)
Provide in Supplementary Information

- [ ] Yes
- [x] No — Explain why this information is not reported/not relevant.

8. Statistics

Number of solar cells tested

- [x] Yes — 56 cells
- [ ] No

Statistical analysis of the device performance

- [x] Yes — Eight solar cells were produced for each condition
- [ ] No

9. Long-term stability analysis

Type of analysis, bias conditions and environmental conditions
For instance: illumination type, temperature, atmosphere humidity, encapsulation method, preconditioning temperature

- [ ] Yes
- [x] No — Explain why this information is not reported/not relevant.
